# CMTM7 recognizes an immune-hot tumor microenvironment and predicts therapeutic response of immunotherapy in breast cancer well

**DOI:** 10.3389/fgene.2022.1051269

**Published:** 2022-12-07

**Authors:** Xingyu Jiang, Zhengtao Qian, Yu Chen, Tao Zhou, Can Zhao, Yongxiang Yin

**Affiliations:** ^1^ Department of Pathology, Wuxi Maternity and Child Health Hospital, Wuxi, China; ^2^ Department of Clinical Laboratory, Changshu Medicine Examination Institute, Changshu, China; ^3^ Research Institute for Reproductive Health and Genetic Diseases, Wuxi Maternity and Child Health Hospital, Wuxi, China; ^4^ Department of Galactophore, Wuxi Maternity and Child Health Hospital, Wuxi, China

**Keywords:** breast cancer, CMTM7, immunotherapy, biomarker, bioinformatics

## Abstract

Breast cancer (BRCA) is a complex disease that leads to major mortalities and unsatisfactory clinical outcomes among women worldwide. CKLF-like MARVEL transmembrane domain-containing 7 (CMTM7) is a potential tumor suppressor and regulator of PD-L1, which has been found as a functional signature in considerable oncogenesis, progression, and therapeutic resistance *via* deletion and downregulation. In this research, triple-negative breast cancer (BRCA), a molecular subtype having a lower response to endocrinotherapy but a higher response to chemotherapy and immunotherapy, showed higher transcriptional levels of CMTM7. Moreover, CMTM7 positively correlated with immunomodulators, tumor-infiltrating immune cells (TIICs), and immune checkpoints in many independent datasets. Furthermore, in an immunotherapy cohort of BRCA, patients with high CMTM7 expression were more sensitive to immunotherapy, and the therapeutic predictive value of CMTM7 is higher than that of PD-1 and PD-L1. To sum up, CMTM7 correlated with an inflamed tumor microenvironment and identified immune-hot tumors, which can be a novel biomarker for the recognition of immunological characteristics and an immunotherapeutic response in BRCA.

## Introduction

Breast cancer (BRCA) is a complex disease that is the cause of major mortalities among women, and the worldwide incidence of BRCA changes between 27 in 100,000 (Africa and East Asia) and 97 in 100,000 (North America) (Bray et al., 2018). According to statistics, BRCA accounts for about 30% of female carcinomas and 15% of mortality-to-incidence (Sung et al., 2021). Although the way BRCA is viewed has changed drastically due to the increasing extensive description of the molecular characteristics of BRCA (Curtis et al., 2012; Ellis et al., 2017), the current therapeutic approaches for BRCA mainly focus on comprehensive treatment, including surgery, chemotherapy, and targeted therapy (Waks and Winer, 2019; Trayes and Cokenakes, 2021). More importantly, controversy remains around all aspects of BRCA treatment (McDonald et al., 2016).

In recent years, with the application of immune-checkpoint inhibitors (ICIs), immunotherapy has developed rapidly and revolutionized the management of multiple solid tumors (Emens, 2018). Accumulating data supports a key role for the immune system in determining both response to standard therapy and long-term survival in patients with BRCA (Savas et al., 2016). For example, a clinical trial on atezolizumab (programmed death 1 [PD-L1] inhibitor) plus nab-paclitaxel in BRCA showed that the median overall survival (OS) in the intention-to-treat patients who received atezolizumab (21 months) was longer than that in those who received a placebo (18.7 months) (Schmid et al., 2020). Although the application of immunotherapy based on PD-1 or PD-L1 blockade has achieved encouraging results, we still cannot ignore that most patients present primary or acquired resistance to immunotherapy agents (Yang, 2015).

Based on previous findings, the composition of the tumor microenvironment (TME) might involve in the response to several treatments. Furthermore, tumors can be identified as cold or hot according to their TME (Cai et al., 2021). To be specific, cold tumors tend to exhibit immunosuppressive TME and are resistant to chemotherapy and immunotherapy, while hot tumors are more sensitive to these therapies and characterized by T-cell infiltration and immunosuppressive TME (Gajewski et al., 2017; Hu et al., 2020; Mao et al., 2022). Collectively, the hot tumors showed a favorite therapeutic response to immunotherapy, such as anti-PD-1/PD-L1 therapy (Zemek et al., 2019). Thus, distinguishing hot and cold tumors is an effective method to demarcate the response to immunotherapy.

Recent in-depth studies revealed that CKLF-like MARVEL transmembrane domain-containing member (CMTM) is closely associated with the genesis, development, and metastasis of tumors, displaying opposing activities in diverse human tumors (Wu et al., 2019). CMTM7 can be a biomarker reflecting the progression and immune status of tumor samples (Jin et al., 2018; Liu et al., 2021). Yongdong Jin et al. (2018) found that the downregulation of CMTM7 facilitates the proliferation and tumorigenesis of gastric cancer cells *in vitro* and *in vivo*. In addition, in BRCA, the downregulation of CMTM7 can activate the EGFR/Akt signaling pathway to promote tumorigenesis and metastasis of tumor cells (Lu et al., 2021). Notably, increasing evidence proved that CMTM6 can maintain the expression of PD-L1 and enhance the ability of the expression of PD-L1 in tumor cells to inhibit T cells (Burr et al., 2017; Mezzadra et al., 2017), and the dual knockdown of CMTM6 and CMTM7 observably downregulated the expression of PD-L1 in the breast cancer cell line MCF-7^Mes^ than the single knockdown of CMTM6 (Xiao et al., 2021). Collectively, all previous findings suggested that CMTM7 could be a biomarker reflecting tumor progression and immune status.

Therefore, in the current study, we first divided the BRCA patients according to the expression levels of CMTM7 and its co-expressed genes. Multiple bioinformatics analyses revealed that patients in the CMTM7-high group tended to have an inflamed TME, which is the characteristic of the immune-hot tumor. Furthermore, in the immunotherapy cohorts of BRCA, patients with the CMTM7-high phenotype showed a better therapeutic response to immunotherapy. In addition, the immunotherapeutic predictive value of CMTM7 is encouragingly higher than that of PD-1 and PD-L1 in BRCA. Our study will provide important information for understanding the significance of CMTM7 in recognizing BRCA patients with immune-hot TME and predicting the therapeutic response of immunotherapy.

## Materials and methods

### Dataset acquisition

The normalized RNA-sequencing profile and clinical annotations of patients in TCGA-BRCA cohort were downloaded from the UCSC Xena website (https://xenabrowser.net/datapages/). In addition, we also obtained the Molecular Taxonomy of Breast Cancer International Consortium (METABRIC) cohort (Curtis et al., 2012) from the cBioPortal for Cancer Genomics (http://cbioportal.org) (Cerami et al., 2012). Furthermore, two immunotherapy cohorts of breast cancer [GSE173839 (Pusztai et al., 2021) and GSE194040 (Wolf et al., 2022)] were also acquired from the Gene Expression Omnibus (GEO) portal (https://www.ncbi.nlm.nih.gov/geo/). For the TCGA-BRCA and METABRIC cohorts, samples with overall survival (OS) above zero days were included in this research. For immunotherapy cohorts, diagnostic patients who received immunotherapy were selected for further analysis.

### Identification of the CKLF-like MARVEL transmembrane domain-containing 7 groups

In order to identify the CMTM7-related groups in TCGA-BRCA cohort, weighted gene co-expression network analysis (WGCNA) was performed to identify genes co-expressed with CMTM7 based on the expression profiles of the top 25% of most variant genes (MVGs) according to the analysis of variance (5,133 genes) first. GS represented the correlation between the gene expression and the CMTM7 transcriptional values. MM represented the correlation between the module eigengene and gene expression. Thus, genes with GS ≥ 0.5 and MM ≥ 0.5 in the module that had the highest correlation with CMTM7 expression values were selected as CMTM7 co-expressed genes. Second, silhouette analysis was used to determine the optimal number of stable groups. The number of clusters corresponding to the maximum silhouette coefficient is the optimal number of stable groups. The R package “ConsensusClusterPlus” is an unsupervised class discovery tool with confidence assessments and item tracking by implementing the consensus clustering method. Therefore, consensus clustering (the “ConsensusClusterPlus” package (Wilkerson and Hayes, 2010) in R with 1,000 iterations and 80% resampling) was performed to determine the optimal number of stable subpopulations based on the expression matrix of 72 CMTM7 co-expressed genes (Table 1). Silhouette analysis and consensus matrixes showed that the optimal number of stable groups was 2. Subsequently, patients in the TCGA-BRCA cohort were divided into two groups by performing consensus clustering at k = 2. Finally, 576 patients were classified as the CMTM7-high group, and 493 patients were classified as the CMTM7-low group.

**TABLE 1:** Co-expressed genes of CMTM7.

List of co-expressed genes of CMTM7					
RARRES1	LOC284578	MIA	KLK8	GPM6B	TCF7L1
CHST3	GABRP	HAPLN3	SFRP1	SOX10	ST8SIA1
C2orf88	KRT16	FAM171A1	TMEM158	LOXL4	SMO
LOC84856	CMTM7	PTX3	KLK10	ROPN1B	TTYH1
C10orf90	RNF175	FERMT1	GSTP1	IRX1	VGLL1
KCNK5	CHODL	PPP1R14C	KCNN4	LEMD1	EPHB1
MFGE8	FOXC1	CA6	C9orf170	RASGEF1C	MOBKL2B
KLK5	FOSL1	NCRNA00092	OSR1	CHI3L1	RGMA
TRPV4	STAC	MMP7	ROPN1	CX3CL1	BCL11A
KLK6	RGS2	HRCT1	PRG2	C6orf15	SOX8
SCRG1	SFRS13B	FAM19A3	GDF5	TMPRSS5	MFI2
WNT6	OCA2	FOLR1	S100B	IL34	CRYAB

### Classification of patients in test datasets

The CMTM7 groups identified in TCGA-BRCA cohort were extrapolated to other datasets (testing datasets) following these steps. First, the gene expression profiles of TCGA-BRCA (training dataset) and test dataset were combined, and the batch effect was removed by the R package “limma” (Ritchie et al., 2015). Subsequently, the classification of patients was predicted by the R package “pamr” according to the nearest shrunken centroids method, an algorithm using “shrunken” centroids as prototypes for each group and identifying the representative genes of each group (Tibshirani et al., 2002).

### Assessment of immunological characteristics of the tumor microenvironment

In order to assess the immunological characteristics of the TME, the ESTIMATE algorithm (Yoshihara et al., 2013), a method inferring tumor purity and stromal and immune cells from tumor samples based on bulk transcriptomic profiles, was performed to assess tumor purity, the ESTIMATE score, the immune score, and the stromal score. The stromal and immune scores were calculated by performing the single-sample gene set enrichment analysis of two user-defined signatures (stromal and immune signatures) (Yoshihara et al., 2013). Based on the stromal and immune scores, the ESTIMATE score was estimated and used to infer the tumor purity (Yoshihara et al., 2013). In addition, the information of 122 immunomodulators including the major histocompatibility complex (MHC), receptors, chemokines, and immune stimulators was collected from the study of Charoentong et al. (2017). To further understand the immunological status of each patient, a set of signature genes for 29 immune cell types and immune-related pathways (Bindea et al., 2013) was used to estimate the infiltration levels of different immune cell populations, and the activities of immune-related pathways and functions of each patient were calculated by using the single-sample gene set enrichment analysis (ssGSEA) in the R package “GSVA” (Ferreira et al., 2021).

### Identification of differentially expressed genes

In order to identify the DEGs for the CMTM7-high and CMTM7-low groups, respectively, the R package “limma” (Ritchie et al., 2015) was used to perform the differential expression analysis. Genes with a fold-change (FC) ≥ 1.5 and adjusted *p*-values < 0.05 were recognized as upregulated genes for the CMTM7-high group, while genes with a FC ≤ −1.5 and adjusted *p*-values < 0.05 were recognized as upregulated genes for the CMTM7-low group.

### Enrichment analysis of gene functions and pathways

Gene Ontology (GO) and Kyoto Encyclopedia of Genes and Genomes (KEGG) pathway enrichment analyses were performed by using the R package “clusterProfile.” The top 10 enriched pathways with the most significant *p*-values were displayed.

### Clinical samples

The BRCA tissue microarray (TMA, Cat. HBreD030CS01) was purchased from Outdo BioTech (Shanghai, China). The HBreD030CS01 microarray contained 30 BRCA samples. Ethical approval for the use of TMAs was granted by the Clinical Research Ethics Committee at Outdo Biotech (Shanghai, China). In addition, five breast fibroma samples were collected by Wuxi Maternity and Child Health Hospital. Ethical approval for the collection of tissue sections was granted by the Clinical Research Ethics Committee, Wuxi Maternity and Child Health Hospital.

### Immunohistochemistry staining and semi-quantitative scoring

IHC staining was conducted on the previous sections according to the standardized procedures (Cai et al., 2021). Sections were retrieved by EDTA (Cat. KGIHC002, KeyGen). The primary antibody used was as follows: anti-CMTM7 (1:200 dilution, Cat. bs-8026R, Bioss). Antibody staining was visualized with DAB and hematoxylin counterstain. The stained sections were independently evaluated by two pathologists. The evaluation standard was on a 12-point scale by calculating the immunoreactivity score (IRS) (Mei et al., 2021a). Briefly, the percentage of positively stained cells was scored from 0 to 4: 0 (<5%), 1 (6%–25%), 2 (26%–50%), 3 (51%–75%), and 4 (>75%). The staining intensity was scored from 0 to 3: 0 (negative), 1 (weak), 2 (moderate), and 3 (strong). The immunoreactivity score (IRS) equals the percentages of positive cells multiplied by staining intensity.

### Statistical analysis

All statistical analyses were handled using R software (version 4.0.4). The significant difference in continuous variables between the two groups was assessed using the Wilcoxon rank-sum test, while Fisher’s exact test was used to measure the difference among categorical variables. Prognostic values were evaluated using the log-rank test. For all analyses, a two-paired *p*-value ≤ 0.05 was deemed to be statistically significant and labeled with *p-value ≤ 0.05, **p-value ≤ 0.01, ***p-value ≤ 0.001, and ****p-value ≤ 0.0001.

## Results

### CKLF-like MARVEL transmembrane domain-containing 7 predicts molecular subtypes in breast cancer

**FIGURE 1:**
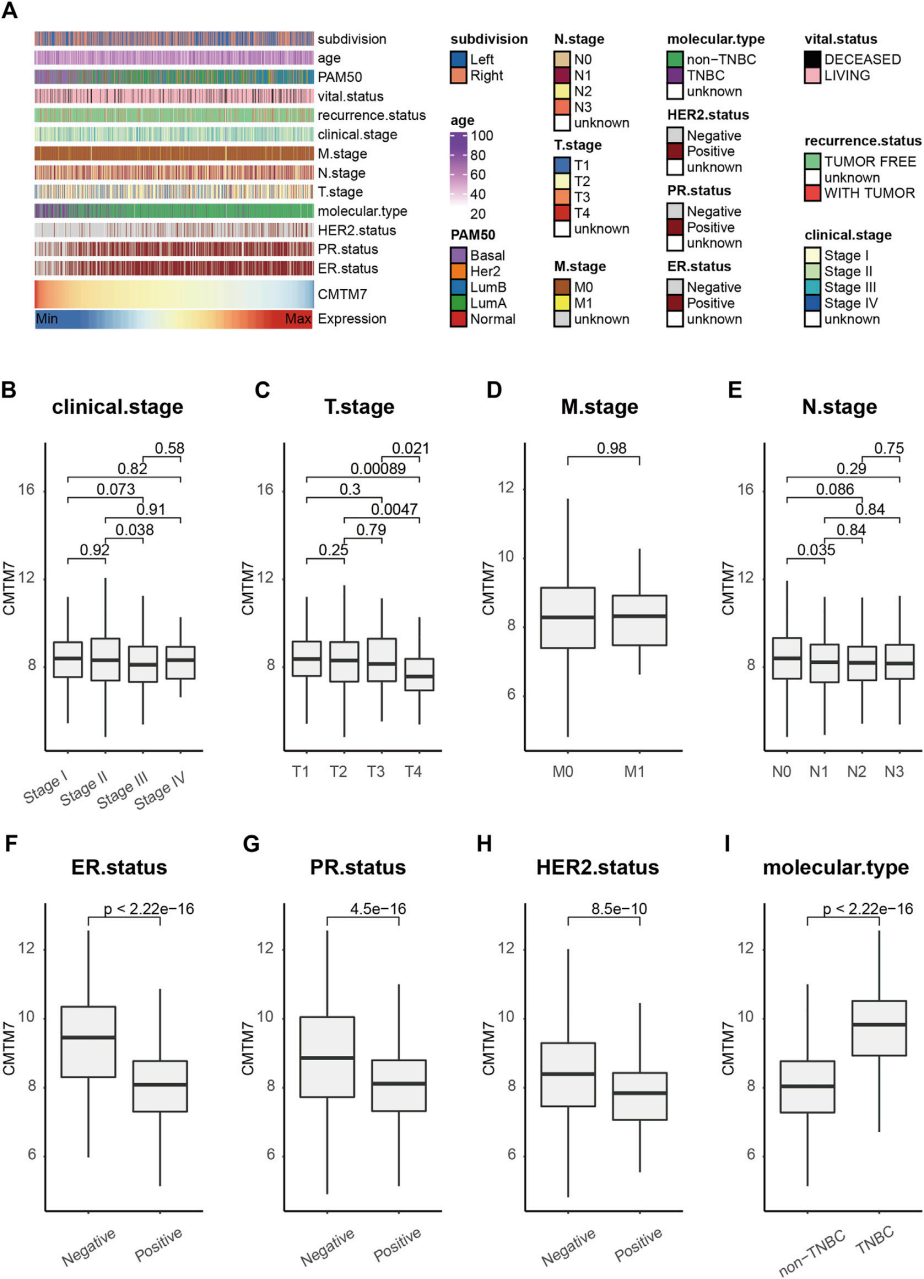
CMTM7 predicts the molecular subtype in BRCA. **(A)** Correlations between CMTM7 and clinicopathological features in BRCA. **(B–I)** Boxplot showing the expression level of CMTM7 among the subtypes of each clinicopathological feature. Horizontal lines in the boxplots represent the median, the lower and upper hinges correspond to the first and third quartiles, respectively, and the whiskers extend from the hinge up to 1.5 times the interquartile range from the hinge. The Wilcoxon rank-sum test was performed to measure the difference between two groups.

Considering that the downregulation of CMTM7 is correlated with tumorigenesis and progression, we first evaluated CMTM7 expression and the clinicopathological features of BRCA. As expected, BRCA patients with a high level of CMTM7 showed significantly better overall survival (log-rank test, *p* = 0.04, Supplementary Figure S1). As shown in Figure 1A, CMTM7 was remarkably associated with previous PAM50 subclasses. Patients with basal-like and normal-like PAM50 phenotypes tended to have higher levels of CMTM7, while other PAM50 subclasses showed lower expression values of CMTM7 (Figure 1A). In addition, there were no significant differences among patients with different pathological characteristics (Figures 1B–E). To be specific, no statistically significant difference in CMTM7 expression among patients with clinical or TNM stages was observed (Figures 1B–E). Last but not least, given the important role of levels of human epidermal growth factor receptor 2 (HER2) and steroid hormone receptors (estrogen receptor [ER] and progesterone receptor [PR]) in subgrouping BRCA patients (Howlader et al., 2014), we next explored the correlation between transcriptional levels of CMTM7 and these receptors. Results showed that CMTM7 was significantly lower in patients with ER-positive (*p* < 0.0001) or PR-positive (*p* < 0.0001) phenotypes (Figures 1A,F,G). In addition, compared with HER2-negative patients, the mRNA expression of CMTM7 was significantly lower in HER2-rich patients (*p* < 0.0001, Figures 1A,H). In addition, CMTM7 expression was remarkably higher in patients with triple-negative breast cancer (TNBC) (*p* < 0.0001, Figures 1A,I), a molecular subtype with dead aggressiveness and a lack of effective therapies (Mei et al., 2020), but often overexpressing PD-L1 and showing encouraging therapeutic responses to immunotherapy (Mei et al., 2021b; Majidpoor and Mortezaee, 2021). Collectively, these results showed that CMTM7 was not associated with pathological stages but can predict the molecular subtypes of BRCA.

### Identification of CKLF-like MARVEL transmembrane domain-containing 7-related breast cancer subtypes

**FIGURE 2:**
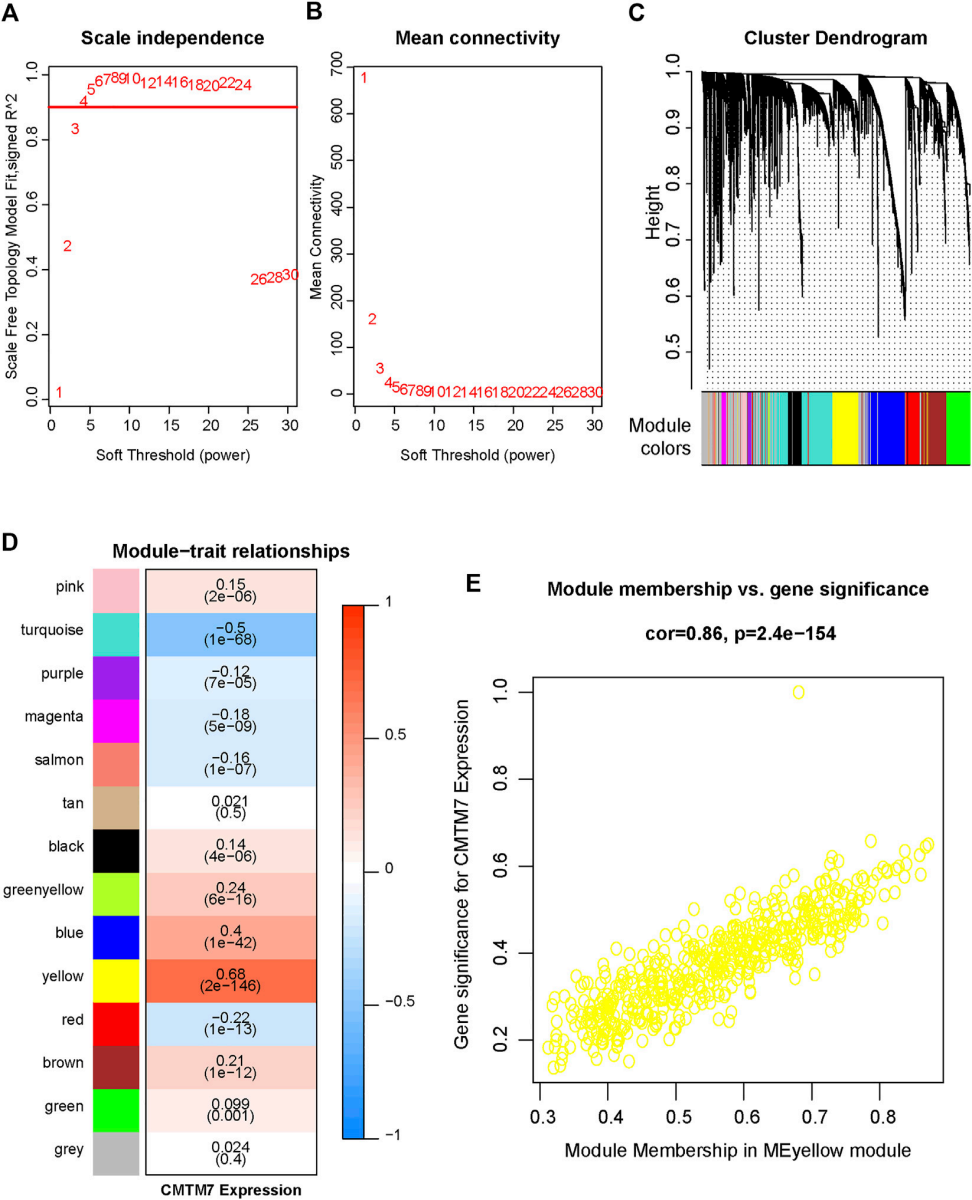
Identification of relevant modules associated with CMTM7 expression in TCGA-BRCA. **(A)** Analysis of the scale-free fitting indices for different soft-thresholding powers (β). **(B)** Mean connectivity analysis of different soft-thresholding powers. **(C)** Clustering dendrograms of genes were based on dissimilarity, topological overlap, and module colors. As a result, 13 co-expressed modules except the gray module were constructed and labeled with different colors. These modules were arranged from large to small according to the number of genes included. **(D)** Heatmap of the correlation between module eigengenes and CMTM7 expression of BRCA. The yellow gene module was revealed to exhibit the highest correlation with CMTM7 expression. **(E)** Scatter plots showing the relationship between MM and GS in the yellow module.

Having observed the value of CMTM7 in predicting the molecular subtypes of BRCA and that it is especially overexpressed in TNBC patients who are relatively sensitive to immunotherapy, we next explored the correlation between CMTM7 expression and immunological characteristics. In order to classify the patients scientifically, we first constructed a WGCNA network by using the R package WGCNA to identify the co-expressed genes of CMTM7 in TCGA-BRCA cohort. In the present research, after constructing a scale-free network based on the soft-thresholding power of β = 4 (scale-free network *R* ^2^ = 0.91, Figures 2A, B), 13 color-coded gene modules except for the gray module were held (Figure 2C). As shown in Figure 2D, the yellow module showed the highest correlation with CMTM7 expression (R = 0.68, *p* < 0.0001, Figure 2D). The GS and MM values for the yellow module in CMTM7 mRNA levels were displayed in scatter plots (Figure 2E). Genes with GS ≥ 0.5 and MM ≥ 0.5 were selected as the co-expressed genes with CMTM7.

**FIGURE 3:**
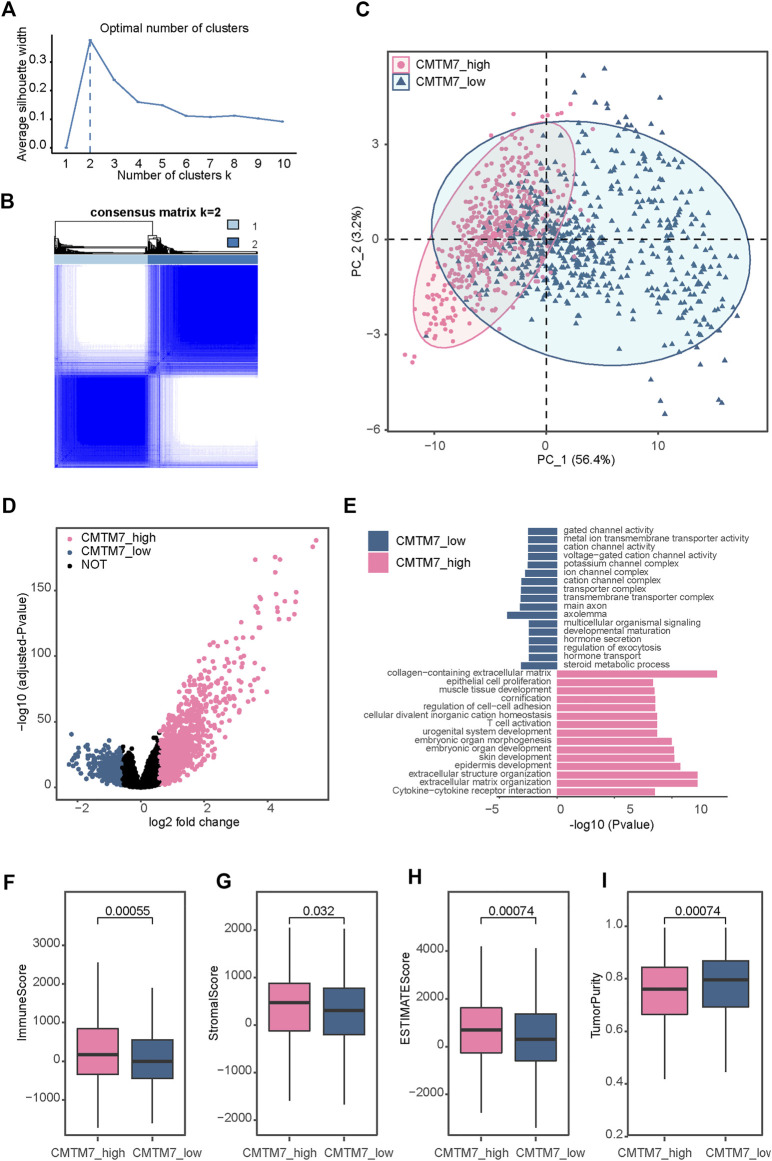
Identification of a CMTM7-related subtype in TCGA-BRCA **(A)** Average silhouette coefficient for k = 1 to k = 10. **(B)** Consensus clustering matrix of TCGA-BRCA samples using the expression matrix of CMTM7 and its co-expressed genes for k = 2. **(C)** Principal component analysis of TCGA-BRCA samples based on the expression matrix of all genes. **(D)** Volcano plot showing the differentially expressed genes (DEGs) for the CMTM7-high and CMTM7-low groups. **(E)** Functional enrichment analysis of DEGs for the CMTM7-high and CMTM7-low groups. **(F–I)** Boxplot showing the immune score, stromal score, ESTIMATE score, and tumor purity between the CMTM7-high and CMTM7-low groups. Horizontal lines in the boxplots represent the median, the lower and upper hinges correspond to the first and third quartiles, respectively, and the whiskers extend from the hinge up to 1.5 times the interquartile range from the hinge. The Wilcoxon rank-sum test was performed to measure the difference between two groups.

Next, we classified the patients in TCGA-BRCA cohort based on the expression matrix of CMTM7 and its co-expressed genes. According to silhouette analysis and consensus matrixes (Figures 3A,B; Supplementary Figure S2), the optimal number of stable groups was 2. Then, 1,069 patients in TCGA-BRCA cohort were divided into two subgroups by performing consensus clustering at k = 2 (Figures 3B,C), including 576 patients in the CMTM7-high group and 493 patients with the CMTM7-low phenotype. Then, DEG analysis was performed to find the specifically expressed genes for the CMTM7-high and CMTM7-low groups, respectively (Figure 3D). Notably, the functional enrichment analysis showed that genes upregulated in the CMTM7-high group were highly related to signaling pathways associated with immune status, such as T-cell activation and cytokine–cytokine receptor interaction (Figure 3E). Consistent with the biological pathways, compared with the CMTM7-low group, patients with the CMTM7-high phenotype showed higher levels of immune score (*p* = 0.00055), stromal score (*p* = 0.032), and ESTIMATE score (*p* = 0.00074) but lower levels of tumor purity (*p* = 0.00074, Figures 3F–I). All the aforementioned results suggested that CMTM7 expression was positively correlated with the inflamed TME.

### Correlation of CKLF-like MARVEL transmembrane domain-containing 7-related breast cancer subtypes with immune infiltration

**FIGURE 4:**
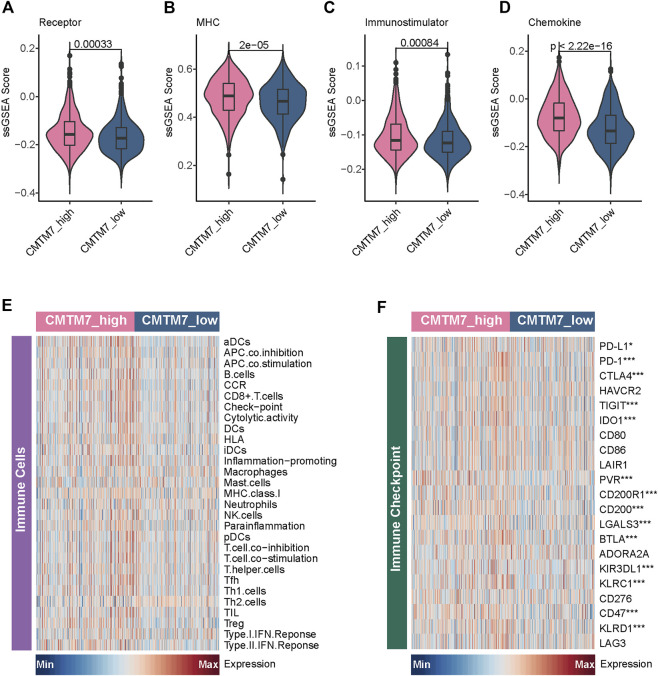
Immunological characteristics between the CMTM7-high and CMTM7-low groups in TCGA-BRCA cohort. **(A–D)** Comparison of the enrichment scores of receptors, MHC, immunostimulators, and chemokines between the CMTM7-high and CMTM7-low groups. **(E)** Heatmap showing the enrichment scores of immune subpopulations and immune-related signaling pathways. **(F)** Heatmap showing the gene expression matrix of immune checkpoint inhibitors.

Given that the patients with the CMTM7-high phenotype tend to have an infiltrating TME and activate some signaling pathways associated with an immune response, we subsequently explored the immunological characteristics between the CMTM7-high and CMTM7-low groups in TCGA-BRCA cohort in depth. As shown in Figures 4A–D, patients with the CMTM7-high phenotype had remarkably higher enrichment scores of chemokines, paired receptors, MHC molecules, and immunomodulators (Figures 4A–D), which were involved in recruiting effector tumor-inflamed immune cells, such as CD8^+^ T cells, macrophages, and antigen-presenting cells. Meanwhile, the CMTM7-high group showed higher enrichment scores of immune-related pathways and a relative abundance of immune cells such as nature killer (NK) cells (Figure 4E), indicating that these patients tend to have more inflamed TME. In addition, the expression of immune checkpoint inhibitors such as PD-1/PD-L1 was reported to be high in inflamed TME (Cai et al., 2021). Consistently, in our research, CMTM7 was found to be positively correlated with a majority of immune checkpoint inhibitors including PD-1, PD-L1, and CTLA4 (Figure 4F).

**FIGURE 5:**
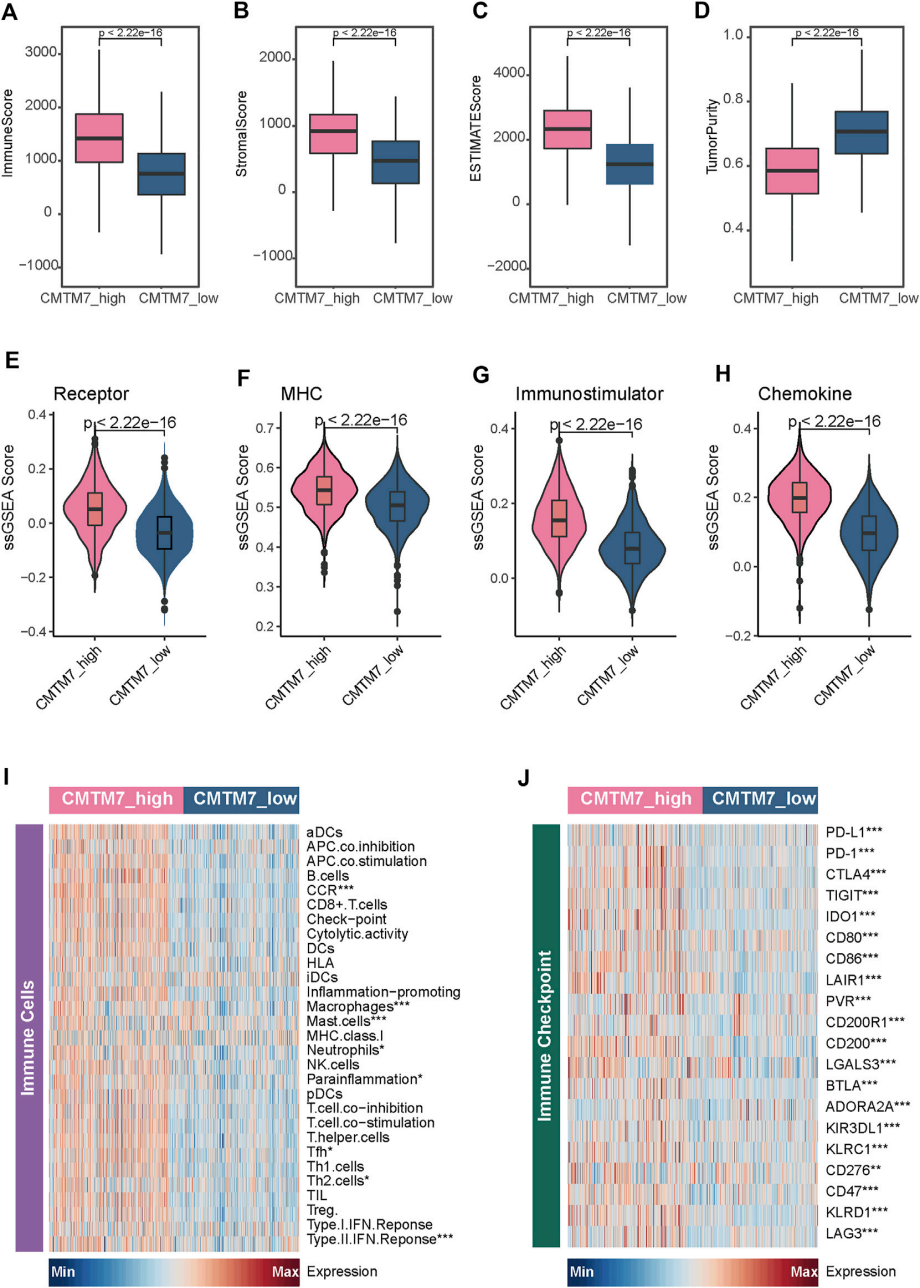
Immunological characteristics between the CMTM7-high and CMTM7-low groups in the METABRIC cohort. **(A–D)** Boxplot showing the immune score, stromal score, ESTIMATE score, and tumor purity between the CMTM7-high and CMTM7-low groups. **(E–H)** Comparison of the enrichment scores of receptors, MHC, immunostimulators, and chemokines between the CMTM7-high and CMTM7-low groups. **(I)** Heatmap showing the enrichment scores of immune subpopulations and immune-related signaling pathways. **(J)** Heatmap showing the gene expression matrix of immune checkpoint inhibitors.

Last but not least, we further validated these findings in the METABRIC cohort. Consistent with the results found in TCGA-BRCA cohort, patients in the CMTM7-high group had remarkably higher levels of immune scores, stromal scores, and ESTIMATE scores, and lower tumor purity (Figures 5A–D). In addition, patients with the CMTM7-high phenotype also showed significantly higher enrichment scores for immunomodulatory factors and immune-related status (Figures 5E–I). Meanwhile, a majority of immune checkpoint inhibitors are also highly expressed in patients in the CMTM7-high group. Totally, CMTM7 is tightly correlated with the development of an inflamed TME, which may play a critical role in identifying the immunogenicity of BRCA.

### CKLF-like MARVEL transmembrane domain-containing 7 predicts the therapeutic response of immunotherapy

**FIGURE 6:**
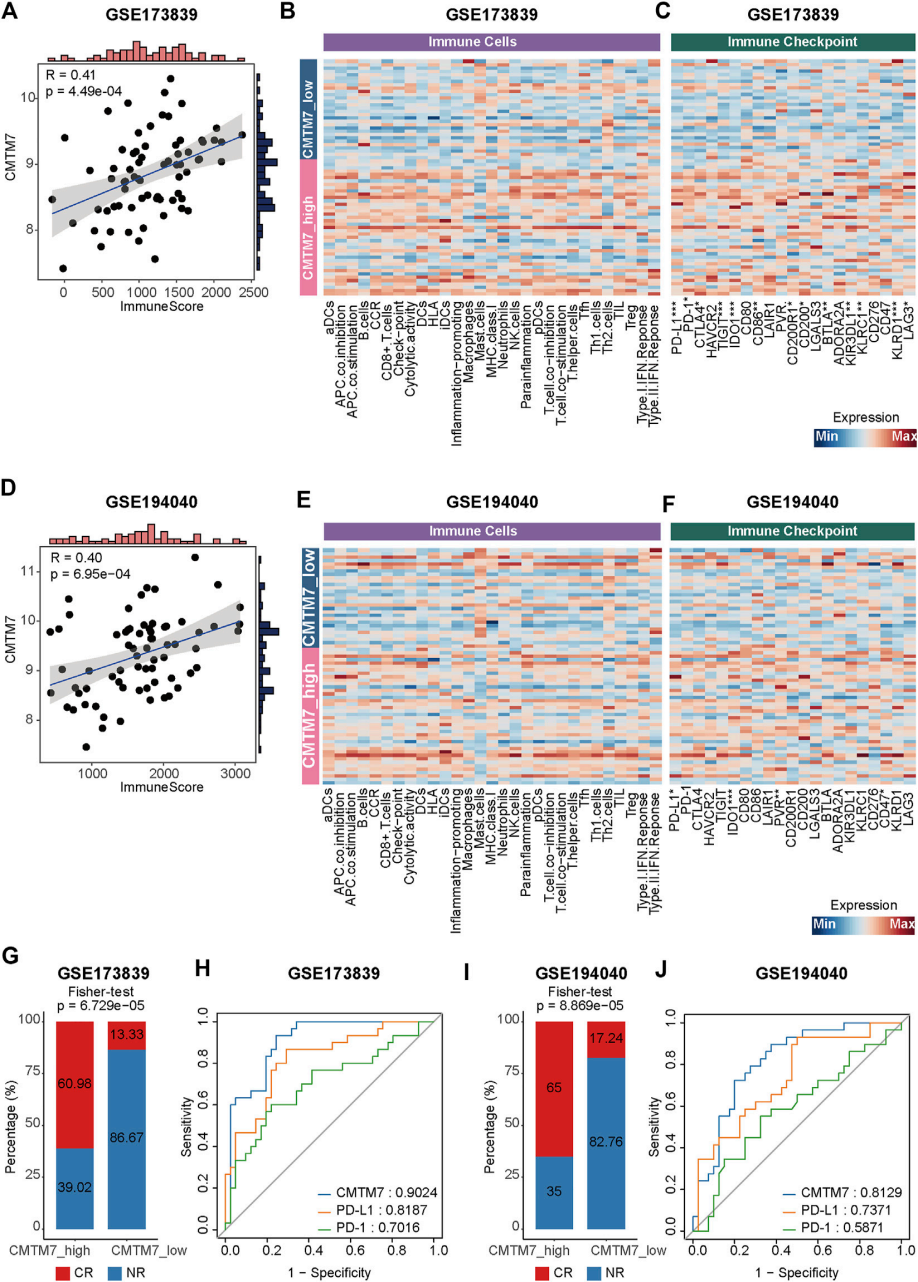
CMTM7 correlated with inflamed TME and predicted the immunotherapeutic response. **(A)** Correlation between CMTM7 expression and immune score in the GSE173839 cohort. **(B)** Heatmap showing the enrichment scores of immune subpopulations and immune-related signaling pathways in GSE173839. **(C)** Heatmap showing the gene expression matrix of immune checkpoint inhibitors in GSE173839. **(D)** Correlation between CMTM7 expression and immune score in the GSE194040 cohort. **(E)** Heatmap showing the enrichment scores of immune subpopulations and immune-related signaling pathways in GSE194040. **(F)** Heatmap showing the gene expression matrix of immune checkpoint inhibitors in GSE194040. **(G)** Barplot showing the percentage of complete remission (CR) and non-complete remission (NR) in the CMTM7-high and CMTM7-low groups in GSE173839. **(H)** ROC curves showing the predictive values of immunotherapeutic response for CMTM7, PD-L1, and PD-1 in the GSE173839 cohort. **(I)** Barplot showing the percentage of CR and NR patients in the CMTM7-high and CMTM7-low groups in the GSE194040 cohort. **(J)** ROC curves showing the predictive values of the immunotherapeutic response for CMTM7, PD-L1, and PD-1 in the GSE194040 cohort.

A previous study found that the dual knockdown of CMTM6 and CMTM7 observably downregulated the expression of PD-L1 in the breast cancer cell line MCF-7^Mes^ (Xiao et al., 2021), indicating the potential role of CMTM7 in reflecting the anti-tumor activity of BRCA. Therefore, we further explored the therapeutic predictive values of CMTM7 in immunotherapy cohorts of BRCA. First, patients in the immunotherapy cohorts were classified into the CMTM7-high and CMTM7-low groups based on the gene expression profiles, respectively (see “Methods”). As expected, the transcriptional levels of CMTM7 were positively correlated with the relative abundance of immune cells in the GSE173839 immunotherapy cohort (*R* ^2^ = 0.41, *p* < 0.0001, Figure 6A). Meanwhile, compared with the CMTM7-low group, patients with the CMTM7-high phenotype had higher levels of inflamed immune cell subpopulations and immune checkpoint inhibitors (Figures 6B, C), in keeping with the results in TCGA-BRCA and METABRIC cohorts. In addition, the results in the GSE194040 cohort were also consistent with the previous results (Figures 6D–F). Encouragingly, patients with a CMTM7-high phenotype were more likely to exhibit sensitivity to immunotherapy (*p* < 0.0001, Figure 6G), and the transcriptional levels of CMTM7 showed more predictive value of an immunotherapy response than those of PD-1 and PD-L1 (CMTM7: AUC = 0.9024; PD-L1: AUC = 0.8187; PDCD1: AUC = 0.7016; Figure 6H). Last but not least, these results were also validated in another immunotherapy cohort for BRCA (Figures 6I, J). Collectively, all the previous results suggested that CMTM7 was positively correlated with immune-hot TME and can be a novel biomarker for predicting the therapeutic response of immunotherapy in breast cancer well.

### Validation of CKLF-like MARVEL transmembrane domain-containing 7 expression in breast cancer and breast fibromas

**FIGURE 7:**
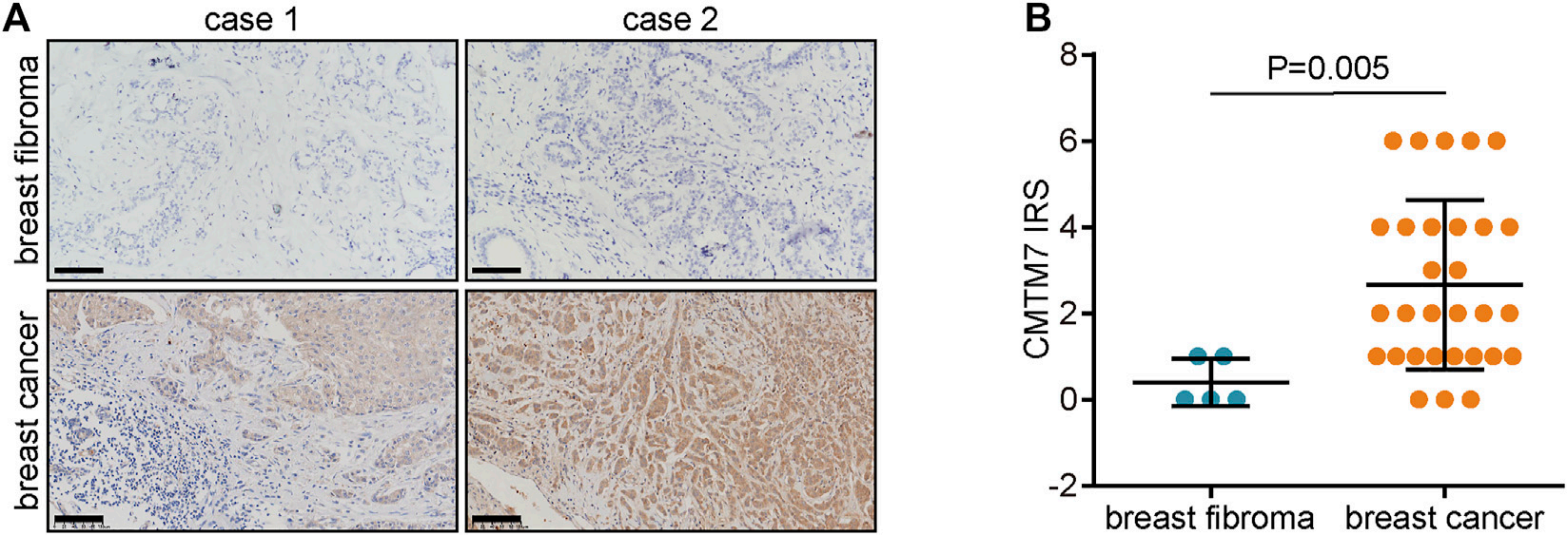
CMTM7 protein expression in BRCA and breast fibromas. **(A)** Representative images uncovering CMTM7 expression in tumor and breast fibroma tissues using anti-CMTM7 staining. **(B)** Semi-quantitative analysis of CMTM7 in tumor and breast fibroma tissues.

To study the protein expression of PSMC2 in BRCA, 30 BRCA samples and five breast fibroma samples were detected by IHC. Figure 7A shows the representative images. The results suggested that CMTM7 expression was significantly enhanced in tumor samples (Figure 7B). Given that CMTM7 is overexpressed in tumor tissues, it could be a candidate target in BRCA.

## Discussion

BRCA is one of the most widespread gynecological oncologies, which leads to major deaths among women around the world (Sung et al., 2021). Although the way BRCA is viewed has changed drastically due to the increasing extensive description of the molecular characteristics of BRCA (Curtis et al., 2012; Ellis et al., 2017), there remains considerable controversy around all aspects of BRCA treatment (McDonald et al., 2016). In addition, although the application of ICIs has revolutionized the management of multiple solid tumors and achieved encouraging therapeutic effects, there are still many patients who present primary and acquired resistance to immunotherapy (Yang, 2015).

According to previous studies, tumors are complex masses consisting of malignant and considerable normal cell subpopulations, such as CD8^+^ T cells and macrophages. The complex interactions among these cells *via* cytokines, chemokines, and growth factors form the TME (Gout et al., 2022). Based on the crosstalk among various cell subpopulations, the TME might be involved in the response to several treatments and the prognosis. Furthermore, tumors can be recognized as cold or hot depending on their TME. To be specific, cold tumors tend to exhibit immunosuppressive TME and are resistant to chemotherapy and immunotherapy, while hot tumors are more sensitive to these therapies, which are characterized by T-cell infiltration and immunosuppressive TME (Cai et al., 2021; Mao et al., 2022). Collectively, the hot tumors showed a favorite therapeutic response to immunotherapy, such as anti-PD-1/PD-L1 therapy. Thus, distinguishing hot and cold tumors is an effective method to demarcate the response to immunotherapy.

Recent in-depth studies revealed that genes belonging to the CMTM family are closely associated with the genesis, development, and metastasis of tumors, displaying opposing activities in diverse human tumors (Wu et al., 2019). As CMTM7 is a potential tumor suppressor and frequently deleted in many carcinomas, its dysfunction could promote oncogenesis and progression in multiple carcinomas (Jin et al., 2018; Lu et al., 2021). Furthermore, the expression of surface PD-L1 in the breast cancer cell line MCF-7^Mes^ was remarkably downregulated *via* the dual knockdown of CMTM6 and CMTM7 compared to that of a single knockdown of CMTM6 (Xiao et al., 2021). Notably, increasing evidence proves that CMTM6, a regulator of PD-L1, can maintain the expression of PD-L1 and enhance the ability of PD-L1 expression in tumor cells to inhibit T cells (Burr et al., 2017; Mezzadra et al., 2017). In addition, the co-expression of CMTM6 and PD-L1 is associated with an active immune microenvironment and a favorable prognosis in colorectal cancer, especially in patients receiving adjuvant chemotherapy (Peng et al., 2021). Furthermore, in triple-negative breast cancer, CMTM6 is positively correlated with PD-L1 and associated with the relapse-free survival rate (Shi et al., 2022). Combined with these findings, CMTM7 might have functions similar to CMTM6, where CMTM7 could be a regulator of PD-L1 and associated with the active immune microenvironment. However, the crucial values of CMTM7 in the recognition of tumor immune status have not been evaluated.

Therefore, in this research, we first reported that the transcriptional levels of CMTM7 were not associated with pathological stages but can predict the molecular subtypes of BRCA. To be specific, patients with negative status for HER2 or steroid hormone receptors showed remarkably higher expression of CMTM7 than those with positive status. Notably, CMTM7 was upregulated in the TNBC subtype of BRCA. As previously reported, TNBC, a molecular subtype with dead aggressiveness and a lack of effective therapies, often overexpressed PD-L1 and showed encouraging therapeutic responses to ICIs.

Given this finding, we next scientifically divided patients into several groups according to CMTM7 expression. First, the WGCNA algorithm was performed to identify the co-expressed genes of CMTM7. Then, patients in TCGA-BRCA cohort were classified into two groups (CMTM7-high and CMTM7-low groups) *via* multiple bioinformatics methods based on the gene expression matrix of these co-expressed genes. Subsequently, the further exploration of immunological characteristics between the CMTM7-high and CMTM7-low groups found that patients with the CMTM7-high phenotype tended to exhibit a more activated immune TME. To be specific, CMTM7 was positively correlated with the enrichment of immunomodulators and TIICs. Notably, the recruitment of effector TIICs was enhanced, thereby promoting the development of an inflamed TME. Meanwhile, we also found that some well-known immune checkpoint inhibitors such as PD-1, PD-L1, and CTLA4 were remarkably highly expressed in the CMTM7-high group. Further analysis in the immunotherapy cohort of BRCA found that high CMTM7 expression was associated with the enhanced response to immunotherapy, and the therapeutic predictive value of it is higher than that of PD-1/PD-L1. Collectively, all our findings suggest that CMTM7 is a novel biomarker that can recognize the immune-hot TME and predict the effective therapeutic response of immunotherapy in BRCA, indicating that measuring the CMTM7 levels of breast cancers could guide the therapeutic schedule.

## Conclusion

To sum up, based on multiple bioinformatics analyses, we reported that the BRCA patients with the CMTM7-high phenotype had the TME with enhanced infiltration of immune cell subpopulations and more activation of immune-related signaling pathways. In addition, patients in the CMTM7-high group were more likely to exhibit sensitivity to immunotherapy. Meanwhile, the transcriptional levels of CMTM7 can predict the immunotherapy response better than those of PD-1 and PD-L1, suggesting that CMTM7 is a novel biomarker that can recognize the immune-hot TME and predict the effective therapeutic response of immunotherapy in BRCA, implying that measuring the CMTM7 levels of breast cancers could guide the therapeutic schedule.

## Data Availability

The original contributions presented in the study are included in the article/Supplementary Material; further inquiries can be directed to the corresponding authors.

## Supplementary material

SUPPLEMENTARY FIGURE S1 Kaplan–Meier analysis of the overall survival of TCGA-BRCA patients. All patients were categorized into two groups based on the median of CMTM7.

SUPPLEMENTARY FIGURE S2 Consensus clustering matrix of TCGA-BRCA samples using the expression matrix of CMTM7 and its co-expressed genes for k = 3 to k = 10.

## References

[B1] Bindea G. Mlecnik B. Tosolini M. Kirilovsky A. Waldner M. Obenauf A. C. (2013). Spatiotemporal dynamics of intratumoral immune cells reveal the immune landscape in human cancer. Immunity 39 (4), 782–795. 10.1016/j.immuni.2013.10.003 24138885

[B2] Bray F. Ferlay J. Soerjomataram I. Siegel R. L. Torre L. A. Jemal A. (2018). Global cancer statistics 2018: GLOBOCAN estimates of incidence and mortality worldwide for 36 cancers in 185 countries. Ca. Cancer J. Clin. 68 (6), 394–424. 10.3322/caac.21492 30207593

[B3] Burr M. L. Sparbier C. E. Chan Y. C. Williamson J. C. Woods K. Beavis P. A. (2017). CMTM6 maintains the expression of PD-L1 and regulates anti-tumour immunity. Nature 549 (7670), 101–105. 10.1038/nature23643 28813417 PMC5706633

[B4] Cai Y. Ji W. Sun C. Xu R. Chen X. Deng Y. (2021). Interferon-induced transmembrane protein 3 shapes an inflamed tumor microenvironment and identifies immuno-hot tumors. Front. Immunol. 12, 704965. 10.3389/fimmu.2021.704965 34456915 PMC8385493

[B5] Cerami E. Gao J. Dogrusoz U. Gross B. E. Sumer S. O. Aksoy B. A. (2012). The cBio cancer genomics portal: An open platform for exploring multidimensional cancer genomics data. Cancer Discov. 2 (5), 401–404. 10.1158/2159-8290.CD-12-0095 22588877 PMC3956037

[B6] Charoentong P. Finotello F. Angelova M. Mayer C. Efremova M. Rieder D. (2017). Pan-cancer immunogenomic analyses reveal genotype-immunophenotype relationships and predictors of response to checkpoint blockade. Cell Rep. 18 (1), 248–262. 10.1016/j.celrep.2016.12.019 28052254

[B7] Curtis C. Shah S. P. Chin S. F. Turashvili G. Rueda O. M. Dunning M. J. (2012). The genomic and transcriptomic architecture of 2, 000 breast tumours reveals novel subgroups. Nature 486 (7403), 346–352. 10.1038/nature10983 22522925 PMC3440846

[B8] Ellis M. J. Suman V. J. Hoog J. Goncalves R. Sanati S. Creighton C. J. (2017). Ki67 proliferation index as a tool for chemotherapy decisions during and after neoadjuvant aromatase inhibitor treatment of breast cancer: Results from the American college of surgeons oncology group Z1031 trial (alliance). J. Clin. Oncol. 35 (10), 1061–1069. 10.1200/JCO.2016.69.4406 28045625 PMC5455353

[B9] Emens L. A. (2018). Breast cancer immunotherapy: Facts and hopes. Clin. Cancer Res. 24 (3), 511–520. 10.1158/1078-0432.CCR-16-3001 28801472 PMC5796849

[B10] Ferreira M. R. Santos G. A. Biagi C. A. Silva Junior W. A. Zambuzzi W. F. (2021). GSVA score reveals molecular signatures from transcriptomes for biomaterials comparison. J. Biomed. Mat. Res. A 109 (6), 1004–1014. 10.1002/jbm.a.37090 32820608

[B11] Gajewski T. F. Corrales L. Williams J. Horton B. Sivan A. Spranger S. (2017). Cancer immunotherapy targets based on understanding the T cell-inflamed versus non-T cell-inflamed tumor microenvironment. Adv. Exp. Med. Biol. 1036, 19–31. 10.1007/978-3-319-67577-0_2 29275462 PMC6693322

[B12] Gout D. Y. Groen L. S. van Egmond M. (2022). The present and future of immunocytokines for cancer treatment. Cell. Mol. Life Sci. 79 (10), 509. 10.1007/s00018-022-04514-9 36066630 PMC9448690

[B13] Howlader N. Altekruse S. F. Li C. I. Chen V. W. Clarke C. A. Ries L. A. (2014). US incidence of breast cancer subtypes defined by joint hormone receptor and HER2 status. J. Natl. Cancer Inst. 106 (5), dju055. 10.1093/jnci/dju055 24777111 PMC4580552

[B14] Hu R. Han Q. Zhang J. (2020). STAT3: A key signaling molecule for converting cold to hot tumors. Cancer Lett. 489, 29–40. 10.1016/j.canlet.2020.05.035 32522692

[B15] Jin Y. Qin X. Jia G. (2018). SOX10-dependent CMTM7 expression inhibits cell proliferation and tumor growth in gastric carcinoma. Biochem. Biophys. Res. Commun. 507 (1-4), 91–99. 10.1016/j.bbrc.2018.10.172 30392914

[B16] Liu B. Lu Y. Zhang T. Yu X. Wang Q. Chi Y. (2021). CMTM7 as a novel molecule of ATG14L-Beclin1-VPS34 complex enhances autophagy by Rab5 to regulate tumorigenicity. Cell Commun. Signal. 19 (1), 77. 10.1186/s12964-021-00720-3 34281589 PMC8287682

[B17] Lu C. Zhao Y. Wang J. Shi W. Dong F. Xin Y. (2021). Breast cancer cell-derived extracellular vesicles transfer miR-182-5p and promote breast carcinogenesis via the CMTM7/EGFR/AKT axis. Mol. Med. 27 (1), 78. 10.1186/s10020-021-00338-8 34294040 PMC8296627

[B18] Majidpoor J. Mortezaee K. (2021). The efficacy of PD-1/PD-L1 blockade in cold cancers and future perspectives. Clin. Immunol. 226, 108707. 10.1016/j.clim.2021.108707 33662590

[B19] Mao W. Cai Y. Chen D. Jiang G. Xu Y. Chen R. (2022). Statin shapes inflamed tumor microenvironment and enhances immune checkpoint blockade in non-small cell lung cancer. JCI Insight 7, e161940. 10.1172/jci.insight.161940 35943796 PMC9675559

[B20] McDonald E. S. Clark A. S. Tchou J. Zhang P. Freedman G. M. (2016). Clinical diagnosis and management of breast cancer. J. Nucl. Med. 57, 9S–16S. 10.2967/jnumed.115.157834 26834110

[B21] Mei J. Hao L. Wang H. Xu R. Liu Y. Zhu Y. (2020). Systematic characterization of non-coding RNAs in triple-negative breast cancer. Cell Prolif. 53 (5), e12801. 10.1111/cpr.12801 32249490 PMC7260065

[B22] Mei J. Liu Y. Yu X. Hao L. Ma T. Zhan Q. (2021). YWHAZ interacts with DAAM1 to promote cell migration in breast cancer. Cell Death Discov. 7 (1), 221. 10.1038/s41420-021-00609-7 34453038 PMC8397740

[B23] Mei J. Xu J. Yang X. Gu D. Zhou W. Wang H. (2021). A comparability study of natural and deglycosylated PD-L1 levels in lung cancer: Evidence from immunohistochemical analysis. Mol. Cancer 20 (1), 11. 10.1186/s12943-020-01304-4 33413365 PMC7789157

[B24] Mezzadra R. Sun C. Jae L. T. Gomez-Eerland R. de Vries E. Wu W. (2017). Identification of CMTM6 and CMTM4 as PD-L1 protein regulators. Nature 549 (7670), 106–110. 10.1038/nature23669 28813410 PMC6333292

[B25] Peng Q. H. Wang C. H. Chen H. M. Zhang R. X. Pan Z. Z. Lu Z. H. (2021). CMTM6 and PD-L1 coexpression is associated with an active immune microenvironment and a favorable prognosis in colorectal cancer. J. Immunother. Cancer 9 (2), e001638. 10.1136/jitc-2020-001638 33579737 PMC7883863

[B26] Pusztai L. Yau C. Wolf D. M. Han H. S. Du L. Wallace A. M. (2021). Durvalumab with olaparib and paclitaxel for high-risk HER2-negative stage II/III breast cancer: Results from the adaptively randomized I-SPY2 trial. Cancer Cell 39 (7), 989–998.e5. e5. 10.1016/j.ccell.2021.05.009 34143979 PMC11064785

[B27] Ritchie M. E. Phipson B. Wu D. Hu Y. Law C. W. Shi W. (2015). Limma powers differential expression analyses for RNA-sequencing and microarray studies. Nucleic Acids Res. 43 (7), e47. 10.1093/nar/gkv007 25605792 PMC4402510

[B28] Savas P. Salgado R. Denkert C. Sotiriou C. Darcy P. K. Smyth M. J. (2016). Clinical relevance of host immunity in breast cancer: From TILs to the clinic. Nat. Rev. Clin. Oncol. 13 (4), 228–241. 10.1038/nrclinonc.2015.215 26667975

[B29] Schmid P. Rugo H. S. Adams S. Schneeweiss A. Barrios C. H. Iwata H. (2020). Atezolizumab plus nab-paclitaxel as first-line treatment for unresectable, locally advanced or metastatic triple-negative breast cancer (IMpassion130): Updated efficacy results from a randomised, double-blind, placebo-controlled, phase 3 trial. Lancet. Oncol. 21 (1), 44–59. 10.1016/S1470-2045(19)30689-8 31786121

[B30] Shi S. Ma H. Y. Sang Y. Z. Ju Y. B. Liu X. Y. Zhang Z. G. (2022). Expression and clinical significance of CMTM6 and PD-L1 in triple-negative breast cancer. Biomed. Res. Int. 2022, 8118909. 10.1155/2022/8118909 35845949 PMC9283057

[B31] Sung H. Ferlay J. Siegel R. L. Laversanne M. Soerjomataram I. Jemal A. (2021). Global cancer statistics 2020: GLOBOCAN estimates of incidence and mortality worldwide for 36 cancers in 185 countries. Ca. Cancer J. Clin. 71 (3), 209–249. 10.3322/caac.21660 33538338

[B32] Tibshirani R. Hastie T. Narasimhan B. Chu G. (2002). Diagnosis of multiple cancer types by shrunken centroids of gene expression. Proc. Natl. Acad. Sci. U. S. A. 99 (10), 6567–6572. 10.1073/pnas.082099299 12011421 PMC124443

[B33] Trayes K. P. Cokenakes S. E. H. (2021). Breast cancer treatment. Am. Fam. Physician 104 (2), 171–178. 34383430

[B34] Waks A. G. Winer E. P. (2019). Breast cancer treatment: A review. JAMA 321 (3), 288–300. 10.1001/jama.2018.19323 30667505

[B35] Wilkerson M. D. Hayes D. N. (2010). ConsensusClusterPlus: A class discovery tool with confidence assessments and item tracking. Bioinformatics 26 (12), 1572–1573. 10.1093/bioinformatics/btq170 20427518 PMC2881355

[B36] Wolf D. M. Yau C. Wulfkuhle J. Brown-Swigart L. Gallagher R. I. Lee P. R. E. (2022). Redefining breast cancer subtypes to guide treatment prioritization and maximize response: Predictive biomarkers across 10 cancer therapies. Cancer Cell 40 (6), 609–623.e6. 10.1016/j.ccell.2022.05.005 35623341 PMC9426306

[B37] Wu K. Li X. Gu H. Yang Q. Liu Y. Wang L. (2019). Research advances in CKLF-like MARVEL transmembrane domain-containing family in non-small cell lung cancer. Int. J. Biol. Sci. 15 (12), 2576–2583. 10.7150/ijbs.33733 31754330 PMC6854381

[B38] Xiao M. Hasmim M. Lequeux A. Moer K. V. Tan T. Z. Gilles C. (2021). Epithelial to mesenchymal transition regulates surface PD-L1 via CMTM6 and CMTM7 induction in breast cancer. Cancers 13 (5), 1165. 10.3390/cancers13051165 33803139 PMC7963182

[B39] Yang Y. (2015). Cancer immunotherapy: Harnessing the immune system to battle cancer. J. Clin. Invest. 125 (9), 3335–3337. 10.1172/JCI83871 26325031 PMC4588312

[B40] Yoshihara K. Shahmoradgoli M. Martinez E. Vegesna R. Kim H. Torres-Garcia W. (2013). Inferring tumour purity and stromal and immune cell admixture from expression data. Nat. Commun. 4, 2612. 10.1038/ncomms3612 24113773 PMC3826632

[B41] Zemek R. M. De Jong E. Chin W. L. Schuster I. S. Fear V. S. Casey T. H. (2019). Sensitization to immune checkpoint blockade through activation of a STAT1/NK axis in the tumor microenvironment. Sci. Transl. Med. 11 (501), eaav7816. 10.1126/scitranslmed.aav7816 31316010

