# Supplementary figures and images for: CMTM7 recognizes an immune-hot tumor microenvironment and predicts therapeutic response of immunotherapy in breast cancer well
Source: Front Genet. 2022 Dec 7;13:1051269. doi: 10.3389/fgene.2022.1051269 (PMC9770089; doi:10.3389/fgene.2022.1051269)

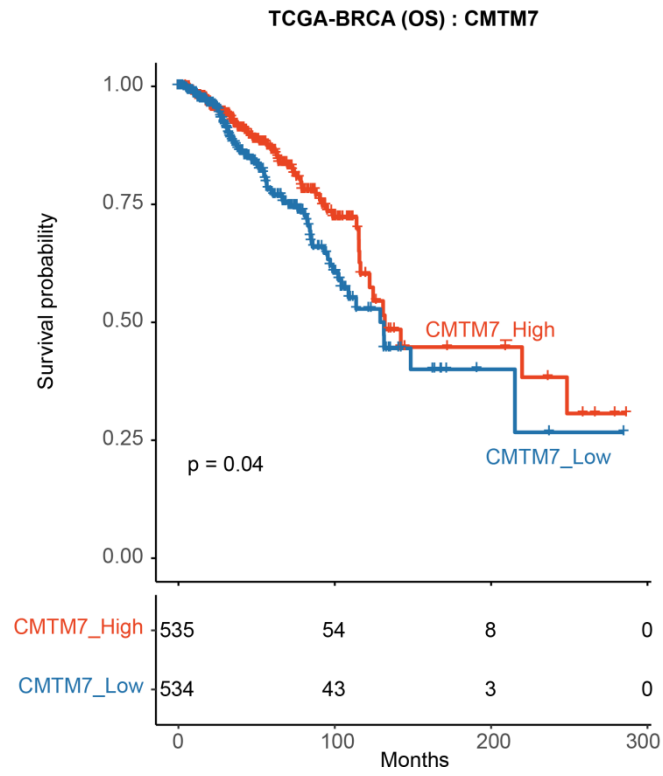

**Figure S1**

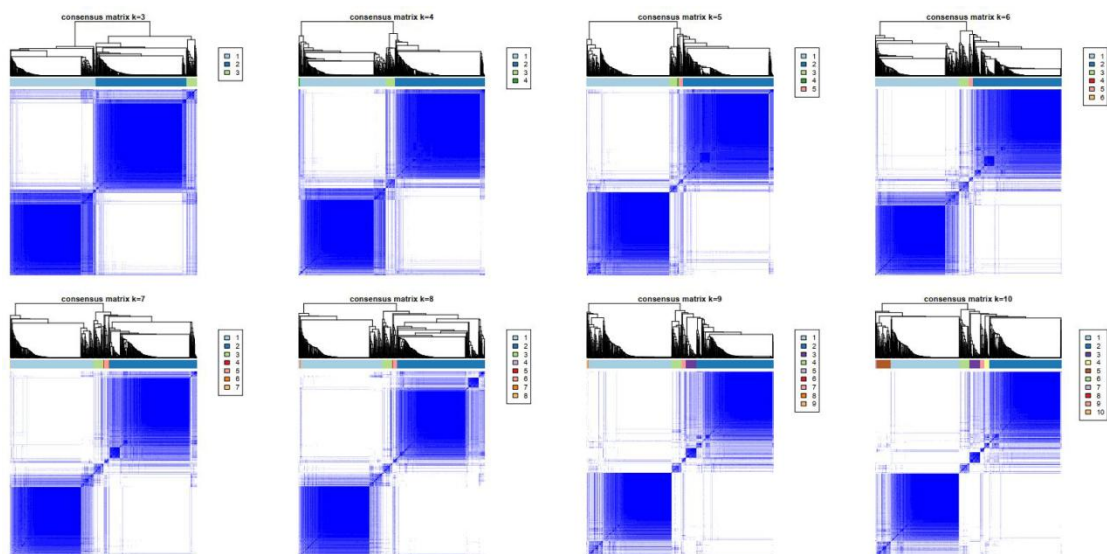

**Figure S2**

Supplement: Supplementary file 1 [file Presentation1.pdf]
